# From gatekeepers to mitochondrial mischief: how bacterial outer membrane proteins crash the host cell party

**DOI:** 10.1093/femsre/fuaf062

**Published:** 2025-12-12

**Authors:** Paloma Osset-Trenor, Markus Proft, Amparo Pascual-Ahuir

**Affiliations:** Grupo de Ingeniería Biomolecular y Biosensores, Centro de Investigación e Innovación en Bioingeniería Ci2B, Universitat Politècnica de València, Ciudad Politécnica de la Innovación, Edificio 8B, Camino de Vera s/n, 46022 Valencia, Spain; Department of Metabolism, Inflammation and Aging, Instituto de Biomedicina de Valencia IBV-CSIC, Consejo Superior de Investigaciones Científicas CSIC, Jaime Roig 11, 46010 Valencia, Spain; Valencia Biomedical Research Foundation, Centro de Investigación Príncipe Felipe (CIPF)—Associated Unit to the Instituto de Biomedicina de Valencia IBV-CSIC, 46012 Valencia, Spain; Department of Metabolism, Inflammation and Aging, Instituto de Biomedicina de Valencia IBV-CSIC, Consejo Superior de Investigaciones Científicas CSIC, Jaime Roig 11, 46010 Valencia, Spain; Valencia Biomedical Research Foundation, Centro de Investigación Príncipe Felipe (CIPF)—Associated Unit to the Instituto de Biomedicina de Valencia IBV-CSIC, 46012 Valencia, Spain; Grupo de Ingeniería Biomolecular y Biosensores, Centro de Investigación e Innovación en Bioingeniería Ci2B, Universitat Politècnica de València, Ciudad Politécnica de la Innovación, Edificio 8B, Camino de Vera s/n, 46022 Valencia, Spain

**Keywords:** outer membrane proteins, bacterial virulence, mitochondrial subversion, β-barrel proteins, apoptosis, outer membrane vesicles

## Abstract

Gram-negative bacteria are equipped with a unique cell envelope structure that includes an outer membrane populated by diverse outer membrane proteins (OMPs). These OMPs are not only essential for bacterial survival, mediating critical functions such as nutrient transport, antibiotic resistance, and structural integrity, but they also play pivotal roles as virulence factors during host-pathogen interactions. Recent research highlights the ability of OMPs to manipulate host cellular processes, often targeting mitochondria to induce cell death or modulate immune responses. This review explores the multifunctional roles of bacterial OMPs, emphasizing their structural features, biogenesis, and pathogenic mechanisms. Furthermore, it delves into how bacterial OMPs exploit host cell machinery, particularly mitochondria, to promote infection, as well as their potential as targets for innovative antimicrobial strategies. Specifically, this review focuses on β-barrel OMPs that reach host mitochondria, detailing their delivery routes and mechanisms of organelle manipulation, while excluding non-β-barrel toxins and secretion-system effectors, to provide a defined perspective on mitochondria-targeting OMP virulence mechanisms.

## Teaser

This review reveals how bacterial outer membrane proteins exploit similarities with mitochondria to hijack host cell functions, highlighting novel insights into bacterial virulence and potential strategies for antimicrobial intervention.

## Introduction

Bacterial outer membrane proteins (OMPs) are integral components of Gram-negative bacterial physiology, serving as versatile mediators of nutrient transport, structural stability, and interactions with the surrounding environment (Vergalli et al. 2020). In pathogenic Gram-negative bacteria, many OMPs have evolved additional functions that contribute to host colonization, immune evasion, and intracellular survival (Lin et al. 2002, Johnson 2018). Their structural versatility, based on β-barrel architectures, underpins their ability to interact with host membranes and subvert cellular machinery (Hermansen et al. 2022). Intriguingly, OMPs are increasingly recognized for their capacity to target mitochondria, exploiting the evolutionary link between mitochondria and bacteria to disrupt or manipulate host cell homeostasis (Marchi et al. 2022).

Mitochondria are multifunctional organelles that integrate metabolic, signaling, and immune pathways (Suomalainen and Nunnari 2024). Beyond their role in ATP production, they act as central hubs for calcium homeostasis, apoptosis, and innate immune signaling, orchestrating the cellular response to infection (Galluzzi et al. 2012, Tiku et al. 2020). These immunometabolic functions make mitochondria both sensors and executors of antibacterial defense. Consequently, numerous bacterial pathogens have evolved mechanisms to manipulate mitochondrial processes to promote their own survival and dissemination (Lobet et al. 2015, Marchi et al. 2022). Among these, specific OMPs have been identified as effectors that target mitochondria, where they can alter organelle integrity and function during pathogenesis. Understanding the dual role of OMPs as both essential bacterial components and sophisticated tools for host manipulation offers valuable insights into bacterial pathogenesis and provides promising avenues for the development of novel therapeutic interventions.

## OMPs: versatile outer membrane proteins of Gram-negative bacteria

Gram-negative bacteria possess a unique cell envelope architecture comprising two membranes: an inner membrane (IM) and an outer membrane (OM), with a hydrophilic periplasmic space containing a thin peptidoglycan layer in between. This arrangement forms the bacterial cell envelope. Whereas IM proteins generally consist of hydrophobic transmembrane α-helices, most OMPs adopt an amphipathic β-sheet structure. These β-sheets fold into a cylindrical β-barrel, which serves as the membrane-spanning domain (Schulz 2000, Montezano et al. 2023). β-barrels can vary greatly in size, with strand numbers ranging from 8 to 36, and are stabilized by hydrogen bonds between their terminal β-strands, forming a highly stable structure (Pautsch and Schulz 1998, Hermansen et al. 2022). Some β-barrel structures are open, allowing for molecular transport (Mayse and Movileanu 2023), while others are occluded by plug domains (Abellon–Ruiz et al. 2023). Certain OMPs also contain periplasmic or extracellular domains linked to the β-barrel (Gruss et al. 2013).

Many OMPs function as oligomers, and both homo-oligomeric and hetero-oligomeric forms of β-barrels have been observed (Meng et al. 2006, Lauber et al. 2018). β-barrel porins form the most abundant protein fraction of the bacterial OM (Vergalli et al. 2020), and their versatile architecture underpins a broad range of functions critical to bacterial survival, such as maintaining cell envelope integrity (Qiao et al. 2014), mediating the selective permeability of nutrients (Ferguson et al. 1998), and contributing to the export of antibiotics and other hydrophobic molecules (Koronakis et al. 2000). OMPs are functionally categorized based on their substrate specificity (Vergalli et al. 2020). Some OMPs function as selective channels, such as LamB, which transports sugars, or Tsx, a nucleoside transporter. Additionally, TonB-dependent transporters, such as FhuA and BtuB, facilitate the uptake of essential metals and vitamins. In contrast, general (or classic) porins allow the nonspecific diffusion of solutes, typically with a molecular mass limit of around 600 Da.

Newly synthesized OMPs in Gram-negative bacteria follow a coordinated pathway for transport and folding (Doyle and Bernstein 2024). This process begins in the cytoplasm, where OMPs are produced with an N-terminal signal sequence that targets them for translocation across the IM. OMPs are first directed to the SecYEG translocon, a protein-conducting channel embedded in the IM. The translocon facilitates the passage of unfolded OMPs across the IM, in a process driven by SecA-dependent ATP hydrolysis and the proton motive force. Once the OMPs are translocated, their signal sequences are cleaved by leader peptidases, leaving the mature proteins in the periplasmic space. In the periplasm, newly translocated OMPs are prone to aggregation, so they are escorted by specific chaperones such as SurA, Skp, and DegP (Devlin and Fleming 2024). These chaperones bind the unfolded OMPs and prevent premature folding, maintaining them in a state suitable for further transport. They also direct the OMPs toward the OM for proper insertion and folding. The final stage of OMP biogenesis occurs at the OM, where the β-barrel assembly machinery (BAM) mediates the insertion and folding of OMPs into their functional β-barrel structures (Wu et al. 2005, Knowles et al. 2009). The BAM complex, consisting of BamA and accessory proteins BamB, BamC, BamD, and BamE, recognizes unfolded OMPs and facilitates their insertion into the OM. BamA, a β-barrel protein itself, is particularly critical in catalyzing the folding of OMPs (Tomasek et al. 2020, Doyle et al. 2022, Shen et al. 2023). This highly regulated process ensures that OMPs are properly inserted and folded, maintaining the integrity and functionality of the bacterial OM. Disruption of the highly conserved BAM complex in Gram-negative bacteria causes OMP folding obstruction, leading to impaired OM function and ultimately, bacterial death. This makes the BAM complex an ideal target for creating novel antibacterial drugs against Gram-negative bacteria (Xu et al. 2023). In addition to the BAM complex, Gram-negative bacteria employ the translocation and assembly module, a related system that assists in the secretion and insertion of certain OMPs, including virulence factors (Selkrig et al. 2012, Wang et al. 2024).

There is a growing body of evidence showing important links between Gram-negative bacterial infections and mitochondrial dysfunction (Marchi et al. 2022), Bacterial effectors, such as OMPs, can modulate mitochondrial function, dynamics, and oxidative stress—processes that are central to both infection outcomes and the development and progression of degenerative pathologies (Bader and Winklhofer 2020, Lotz et al. 2021, Zheng et al. 2025).

To understand how bacterial β-barrel OMPs hijack mitochondria, it is first necessary to consider their evolutionary and structural counterparts within the organelle itself. The structural and mechanistic parallels between these mitochondrial and bacterial β-barrels provide a framework for explaining how bacterial OMPs can engage or disrupt mitochondrial membranes during infection.

## Functions of mitochondrial β-barrel proteins in eukaryotic cells

**Figure 1.**
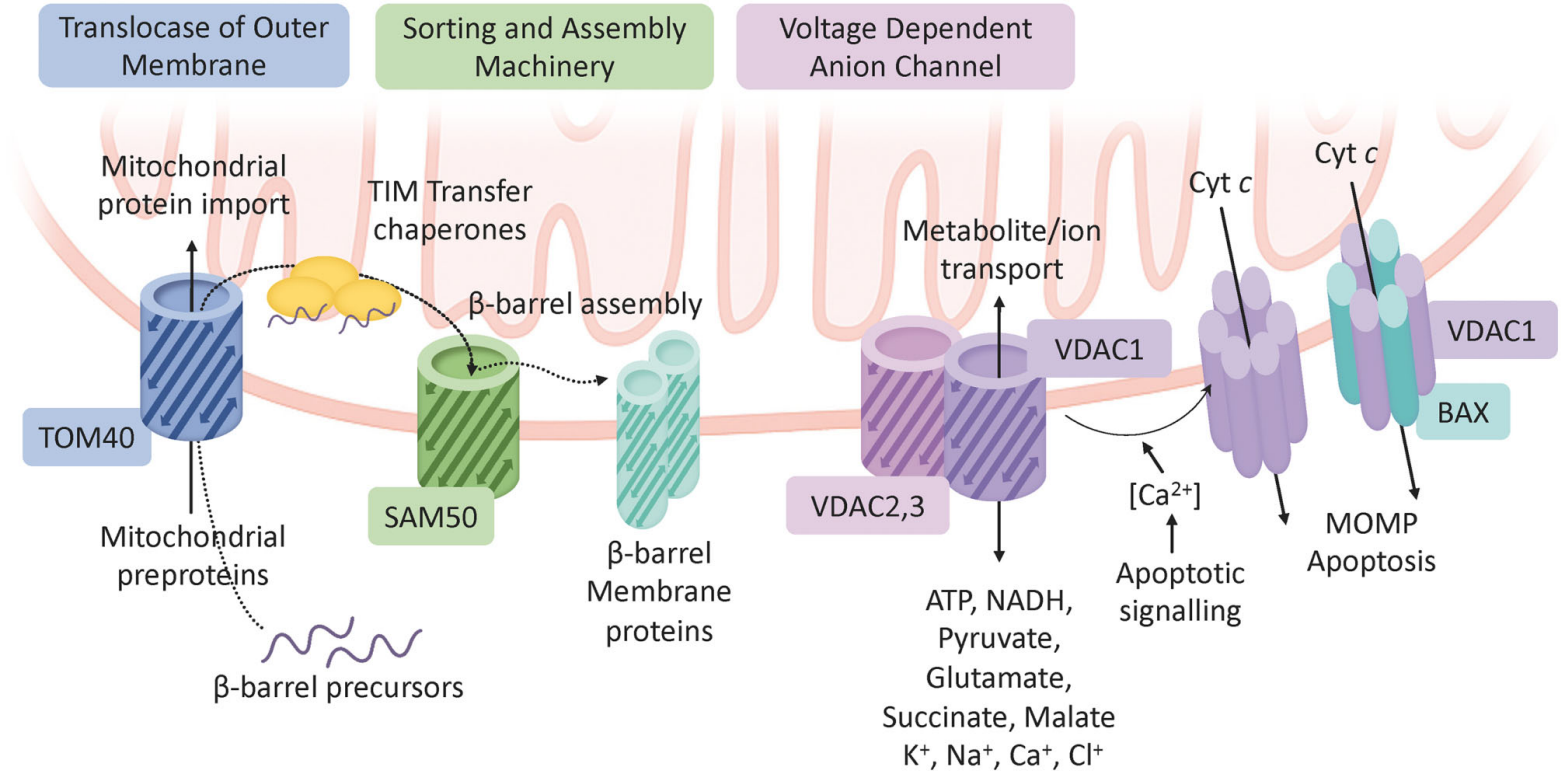
Functions of eukaryotic β-barrel proteins of the outer mitochondrial membrane (OMM). TOM40 serves as the central channel protein of the TOM complex, facilitating the import of nuclear-encoded mitochondrial proteins. β-barrel precursors are guided by TIM transfer chaperones to the SAM complex. SAM50 is the core component of the SAM complex, responsible for the assembly of β-barrel proteins in the OMM. VDAC1, VDAC2, and VDAC3 function as voltage-dependent anion channels (VDACs), enabling the transport of metabolites and ions across the OMM. VDAC1 can undergo apoptotic transformation into oligomeric pores, which may incorporate the pro-apoptotic Bax protein, leading to mitochondrial outer membrane permeabilization (MOMP), cytochrome *c* (Cyt *c*) release, and apoptotic cell death.

Mitochondria arose from an ancestral alphaproteobacterium through endosymbiosis (Gray et al. 1999, Fan et al. 2020), retaining characteristic β-barrel proteins in their OM—an evolutionary feature shared exclusively with Gram-negative bacteria (Roumia et al. 2020). In human mitochondria, only three β-barrel proteins exist: TOMM40, SAMM50, and VDAC1,2,3 (Fig. 1). These proteins are central to mitochondrial homeostasis and exemplify how bacterial structural motifs have been conserved for organelle biogenesis and signaling.

TOMM40 forms the core channel of the translocase of the outer membrane (TOM) complex, mediating the import of most nuclear-encoded mitochondrial proteins (Araiso et al. 2022). SAMM50 is the β-barrel component of the sorting and assembly machinery (SAM), which inserts β-barrel proteins into the OMM (Ganesan et al. 2024). The SAM complex is evolutionarily related to the bacterial BAM complex, illustrating a direct continuity between bacterial and mitochondrial membrane biogenesis (see Box 1), with a significant portion of the OMM still composed of β-barrel proteins (Morgenstern et al. 2021).

Box 1. Biogenesis of mitochondrial β-barrel proteins: a conserved bacterial legacy

Mitochondrial β-barrel proteins share an evolutionary origin with bacterial OMPs and are assembled through a conserved pathway that reflects their bacterial ancestry. In human mitochondria, these proteins—including TOMM40, SAMM50, and the VDACs (Fig. 1)—are encoded by nuclear genes and synthesized in the cytosol as precursor polypeptides bearing targeting elements that direct them to the mitochondrial surface (Ganesan et al. 2024). The molecular principles of β-barrel protein biogenesis have been elucidated primarily through studies in fungal mitochondria. Upon reaching the organelle, the precursors engage the TOM complex, in which TOM40 constitutes the central import pore. The TOM complex serves as the principal entry gate for almost all mitochondrial proteins and mediates the passage of β-barrel precursors into the intermembrane space (IMS) (Araiso et al. 2022).

Within the IMS, a specialized set of small TIM chaperones binds the hydrophobic precursors, preventing their aggregation and escorting them toward the assembly site at the OMM (Diederichs et al. 2021). Integration into the membrane is catalyzed by the SAM complex, whose core subunit, SAM50, belongs to the Omp85 superfamily and is evolutionarily related to the bacterial BamA protein (Heinz and Lithgow 2014, Diederichs et al. 2021). SAM50 does not act alone but assembles with two peripheral membrane proteins, SAM35 and SAM37, which are unrelated to the bacterial BamB–E proteins, illustrating both conservation and divergence of β-barrel assembly pathways across evolution (Diederichs et al. 2020). The SAM complex recognizes conserved β-signal motifs near the C-terminus of substrate proteins and mediates their stepwise folding and insertion into the OMM to form mature β-barrels (Takeda et al. 2023).

Beyond its catalytic role in membrane insertion, SAM50 also contributes to the structural organization of the OMM, establishing physical and functional contacts with other mitochondrial machineries such as the TOM complex and the MICOS system (Ganesan et al. 2024). Loss or depletion of SAM components leads to defective β-barrel assembly, compromised mitochondrial morphology, and impaired bioenergetic function (Ganesan et al. 2024).

Despite the spatial and compositional divergence between bacteria and mitochondria, the fundamental logic of β-barrel protein biogenesis remains conserved. In both systems, unfolded precursors traverse a hydrophilic compartment—the periplasm in bacteria or the IMS in mitochondria—are stabilized by dedicated chaperones, and are ultimately folded and inserted into the OMM by members of the Omp85 translocase family (Walther, Rapaport et al. 2009). This evolutionary continuity illustrates how the OMM retains core features of its bacterial ancestor and provides a mechanistic basis for the capacity of certain bacterial β-barrel effectors to mimic or interfere with host mitochondrial pathways.

Among these proteins, VDAC plays the most dynamic physiological role. Representing up to 35% of the total OM area, VDAC governs the exchange of ions and metabolites and participates in apoptosis through controlled pore formation (Fuchs et al. 2020, Shoshan–Barmatz et al. 2020, Belosludtseva et al. 2024). VDAC oligomerization can release cytochrome c and AIF (Apoptosis inducing factor), triggering caspase-dependent or -independent cell death (Magrì et al. 2018). Interactions with pro- and anti-apoptotic BCL-2 family proteins (BAX, BAK, and VDAC2) fine-tune this process (Cheng et al. 2003, Czabotar and Garcia–Saez 2023).

However, it is important to note that the pro-apoptotic BCL-2 family proteins BAX and BAK are generally considered both necessary and sufficient for MOMP and the consequent release of cytochrome c (Czabotar and Garcia–Saez 2023, Glover et al. 2024). Upon activation, BAX and BAK undergo conformational changes and oligomerize within the OMM to form proteolipid pores that allow intermembrane-space proteins to escape into the cytosol, committing the cell to apoptosis. This mechanism is regarded as the canonical and essential pathway for MOMP in mammalian cells. In contrast, VDAC oligomerization should be viewed as a modulatory rather than a primary mechanism of MOMP (Flores–Romero et al. 2023). Several studies indicate that VDAC may influence apoptotic sensitivity by regulating ion and metabolite fluxes, calcium buffering, or reactive oxygen species (ROS) generation, thereby indirectly affecting BAX/BAK activation or mitochondrial membrane potential (Tan and Colombini 2007, Tomasello et al. 2009, Shoshan–Barmatz et al. 2020).

The structural and mechanistic parallels between mitochondrial and bacterial β-barrels explain why bacterial OMPs can mimic, integrate into, or highjack mitochondrial membranes (see Box 1). These evolutionary echoes underpin the pathogenic strategies discussed in the following sections, where PorB, OmpA variants, and related OMPs exploit mitochondrial import routes and signaling nodes to modulate apoptosis and mitophagy.

## How bacterial OMP virulence factors manipulate host cell physiology

Although mitochondria originated from an ancient endosymbiotic event over 1.45 billion years ago (Gray et al. 1999, Fan et al. 2020), they remain central to eukaryotic cell function, governing essential processes necessary for maintaining cellular and organismal balance. Mitochondria play a critical role in regulating intracellular calcium levels and house specialized molecular mechanisms that trigger regulated cell death, either through apoptosis or necrosis driven by mitochondrial permeability transition (Glover et al. 2024). Due to mitochondria’s critical role in regulating host responses, including defense against bacterial infections and innate immunity (Galluzzi et al. 2012, Tiku et al. 2020), many intracellular bacteria have evolved strategies to exploit mitochondrial functions for their own benefit. Likewise, extracellular bacteria have developed mechanisms to target mitochondria, inducing in many occasions cell death to access nutrients and create favorable conditions for their survival, replication and dissemination (Marchi et al. 2022).

### Transmission of β-barrel virulence factors into host cells

The ability of a pathogenic bacterium to successfully infect a host and cause disease symptoms relies on the coordinated activity of specific virulence factors (Johnson 2018). Their functions include motility and chemotaxis to locate a suitable environment, adherence to host surfaces, the ability to cross epithelial barriers, evading host immune responses and manipulating host cell homeostasis and metabolism, and toxin production. Most virulence factors are proteins that are either displayed on the bacterial surface or secreted externally.

Unlike classical protein effectors secreted through type III or IV secretion systems, or soluble exotoxins that act from the extracellular space, β-barrel OMPs represent a structurally and mechanistically distinct virulence paradigm. Their stable β-sheet architecture enables autonomous folding within lipid bilayers, allowing them to remain functional outside the bacterium and even after delivery via outer membrane vesicles (OMVs) (Kuehn and Kesty 2005, Schwechheimer and Kuehn 2015). This membrane-embedded configuration distinguishes OMPs from translocated effectors that depend on specialized injectisome machineries (Cornelis 2006, Christie et al. 2014). Moreover, several β-barrel OMPs exploit the host mitochondrial import machinery—particularly the TOM complex—by mimicking conserved β-signal motifs shared with endogenous mitochondrial proteins (Kutik et al. 2008, Kreimendahl et al. 2020). These unique features enable OMPs to cross biological membranes independently, integrate into host organelles, and directly modulate mitochondrial physiology, setting them apart from conventional bacterial secretion-system effectors or soluble toxins.

In Gram-negative bacteria, exporting proteins—including virulence factors—is a complex process that relies on specialized secretion systems (Costa et al. 2015). While some virulence factors are released directly via one-step mechanisms such as Type I, III, and IV secretion systems, others—including many OMPs—follow two-step pathways, reaching the periplasm before being inserted into the OM or secreted externally through Type II and Type V systems (Costa et al. 2015, Green and Mecsas 2016).

In Gram-negative bacteria, β-barrel virulence factors that follow two-step secretion pathways are first transported across the IM via the Sec system and then inserted into the OM by BAMs. Among these, the Type V secretion systems (T5SS) are remarkable because their substrates—known as autotransporters—combine a β-barrel domain embedded in the outer membrane with an N-terminal passenger domain that is translocated to the bacterial surface, often conferring virulence functions (Meuskens et al. 2019, Dautin 2021). Although traditionally described as “self-translocating,” it is now recognized that the passenger domain is secreted through a hybrid channel formed by the β-barrel of the autotransporter and the BamA barrel, rather than through its own pore (Doyle and Bernstein 2021). Thus, the term autotransporter is a historical misnomer. T5SS has several subtypes (Va–Vf), including systems used by *Neisseria gonorrhoeae, Shigella flexneri*, and *Helicobacter pylori* (Pohlner et al. 1987, Brandon et al. 2003, Chauhan et al. 2019).

An independent way of transmission of pathogenicity factors has been defined with the discovery of OMVs (Schwechheimer and Kuehn 2015, Magaña et al. 2023). OMVs are small, spherical, bilayered particles with diameters between 20 and 350 nm that are released during the growth phases of Gram-negative bacteria. OMVs have been detected across all studied Gram-negative species, including *Pseudomonas aeruginosa, Vibrio cholerae, N. meningitidis, N. gonorrhoeae, Salmonella enterica, Acinetobacter baumannii, He. pylori*, and *Haemophilus influenzae*.

The OM structure, composed of phospholipids in the inner leaflet and lipopolysaccharides (LPS) in the outer leaflet, and the periplasm containing a wide variety of proteins, is crucial to preserving high concentrations of cargoes such as OMPs and protecting them from degradation. When the OMVs are expelled, they carry a diverse array of biomolecules, with particularly high concentrations of toxins and virulence factors that facilitate communication with host cells and can initiate pathogenic processes, especially mitochondrial dysfunction and apoptotic cell death, independently of live bacterial cells (Ellis and Kuehn 2010, Toyofuku et al. 2019, Magaña et al. 2023). The marked diversity of OMVs across microorganisms suggests that their biogenesis involves specific sorting mechanisms, rather than being a by-product of cell decay. This view supports OMV formation as an active, regulated process central to bacterial physiology and pathogenesis (Sartorio et al. 2021).

The molecular cargo of OMVs can vary depending on the bacterial type, growth conditions, and environmental factors. Proteins constitute one of the most abundant components in OMVs and play diverse functional roles. The emergence of technologies such as mass spectrometry over the last decade has enabled the identification of thousands of OMV-associated proteins, and OMVs of clinically relevant bacteria are highly enriched in OMPs, such as OmpA, OmpC, OmpF, PorB, and autotransporters, many of which are virulence factors themselves (Mullaney et al. 2009, Mendez et al. 2012, Toyofuku et al. 2019, Sartorio et al. 2021, Dhital et al. 2024). OMVs preserve the native lipid environment of the bacterial OM allowing β-barrel OMPs to remain folded and frequently oligomeric rather than denatured. Structural and biophysical studies demonstrate that porins such as PorB and OmpF are exported in OMVs as pre-assembled oligomers that retain conformational integrity and channel activity (Deo et al. 2018, Donnarumma et al. 2018). High-resolution imaging further reveals OMP-dense microdomains within OMV membranes (Manioglu et al. 2023), underscoring that vesiculation maintains native OMP organization and enables functional delivery of these membrane-embedded virulence factors.

Mechanistically, OMVs can fuse directly with host plasma membranes or enter cells through endocytic routes such as clathrin-mediated uptake, caveolin-dependent endocytosis, or macropinocytosis (O’Donoghue and Krachler 2016). Once internalized, OMV cargo can escape endolysosomal degradation through pH buffering by LPS and by fusion with early endosomes, enabling the translocation of stable OMP oligomers into the cytosol and subsequently to mitochondria, as has been reported for PorB (Deo et al. 2018). Box 2 summarizes briefly the internalization routes and proteostatic checkpoints of OMVs targeting mitochondria. Because many of these mechanistic insights derive from studies employing recombinant OMPs or purified OMVs, it is essential to recognize the methodological advantages and limitations of these experimental systems. Such approaches have been instrumental in defining mitochondrial targeting and import pathways but are also prone to artifacts if folding state, purity, or membrane specificity are not rigorously controlled. Box 3 summarizes key methodological considerations and validation criteria to ensure reliable interpretation of OMP–mitochondria interaction studies.

Box 2. Delivery routes and proteostasis checkpoints from OMVs to mitochondria

OMVs serve as effective long-distance delivery vehicles for β-barrel OMPs and other virulence factors from Gram-negative bacteria to host cells. After release, OMVs adhere to host membranes through electrostatic interactions, lipid–lipid contacts, or receptor binding, and enter cells by multiple pathways, including clathrin- and caveolin-mediated endocytosis, macropinocytosis, or lipid raft-dependent uptake (Schwechheimer and Kuehn 2015, O’Donoghue and Krachler 2016). Once internalized, OMVs traffic through the endosomal system, where cargo fates diverge, most vesicles undergo lysosomal degradation, but a subset can escape into the cytosol, aided by fusogenic lipids or transient endosomal rupture (Caruana and Walper 2020, Ribovski et al. 2023, Chatterjee et al. 2024).

Within the cytosol, OMV cargo proteins encounter a series of proteostatic barriers that determine whether they are degraded or can reach mitochondria. Host chaperones, proteasomes, and autophagy pathways recognize misfolded or foreign β-barrels, restricting their accumulation (Bardoel and Strijp 2011, Riebisch et al. 2021). However, stable or oligomeric OMPs that resist turnover may engage mitochondrial import machineries such as the TOM complex, exploiting, e.g. residual β-signal motifs for insertion or interaction with the OM. This defines a sequential route: OMV release—endocytic uptake—endosomal escape—cytosolic surveillance—mitochondrial targeting, in which each step represents a checkpoint balancing immune detection, proteostasis control, and potential mitochondrial subversion.

Box 3. Methodological considerations for studying OMP-mediated mitochondrial subversion using recombinant proteins and OMVs

Recombinant OMPs and purified OMVs are indispensable tools for dissecting bacterial strategies that target host mitochondria. Their use enables precise mechanistic analyses—such as mutational dissection, dose–response experiments, and kinetic tracking of mitochondrial perturbations—without the confounding variables of live infection. OMVs, in particular, preserve OMPs in their native lipid and oligomeric states, maintaining physiological folding and channel activity (Schwechheimer and Kuehn 2015, Lei et al. 2024). However, both systems present methodological challenges that require careful validation to ensure specificity and reproducibility.

A frequent pitfall arises from endotoxin carryover. Even highly purified OMPs and OMVs can retain lipopolysaccharides, lipoproteins, or periplasmic contaminants that trigger stress responses or nonspecific membrane effects. To control for these artifacts, experimental protocols typically include enzymatic lipopolysaccharide removal using, e.g. polymyxin B treatment, Triton X-114 phase separation, or ReLipopolysaccharide (Re-LPS) depletion assays, followed by Limulus Amebocyte Lysate testing to confirm residual endotoxin levels (Petsch and Anspach 2000, Hirayama and Sakata 2002, Chen et al. 2025). These measures are essential to ensure that observed cellular effects truly result from OMP or OMV components rather than contaminating endotoxins.

Proper protein folding and oligomerization must also be confirmed, as misfolded β-barrel monomers or detergent micelles can damage membranes nonspecifically. To ensure structural integrity, recombinant OMPs are typically reconstituted into lipid vesicles or detergent systems that preserve their native β-barrel conformation, and folding is verified by biophysical assays such as circular dichroism spectroscopy, limited proteolysis, or heat-modifiability in sodium dodecyl sulfate-polyacrylamide gel electrophoresis (SDS-PAGE) (Kleinschmidt 2015). Functional oligomerization can be further assessed by blue-native or semi-native PAGE (Morales et al. 2024). These validation steps are critical to distinguish physiological OMP activity from nonspecific membrane disruption due to misfolded protein or detergent artifacts.

Concentration-dependent artifacts are another major concern: high micromolar doses or residual detergents may compromise membranes irrespective of specific import. Experiments should therefore include a full dose–response analysis with vehicle and inactive-mutant controls, reporting both protein-to-cell ratios and detergent residuals. Genuine effects are typically observed at sub-lytic concentrations and absent in vehicle-only conditions.

When working with OMVs, heterogeneity in vesicle size, composition, or uptake route can greatly influence outcomes (Bitto and Kaparakis-Liaskos 2022, Castillo–Romero et al. 2023). OMVs should be characterized by nanoparticle tracking analysis, dynamic light scattering, and/or electron microscopy to confirm homogeneity (Schwechheimer and Kuehn 2015, Klimentová and Stulík 2015). Protein, lipid, and lipopolysaccharide content should be quantified, and uptake mechanisms defined using inhibitors of clathrin- or caveolin-mediated endocytosis, as well as endosomal pH modulation (O’Donoghue and Krachler 2016). Direct evidence of mitochondrial delivery can be obtained by fluorescent cargo tracking or subcellular fractionation.

Ensuring mitochondrial fraction purity is critical, since crude isolates often contain ER or lysosomal contaminants that can confound localization studies. High-quality preparations require density-gradient purification and immunoblot validation using marker proteins—TOM20 or VDAC for mitochondria, Calnexin for endoplasmatic reticulum, LAMP1 for lysosomes, and Na⁺/K⁺-ATPase for plasma membrane (Wieckowski et al. 2009). A strong enrichment of mitochondrial markers with minimal contamination provides confidence in localization data.

True mitochondrial membrane integration must be distinguished from peripheral binding. This is achieved by protease-protection and alkaline-extraction assays: OMPs correctly inserted into the OMM remain protease-protected but become accessible upon detergent lysis, and resist extraction at alkaline pH 10.8–11.5 (Kutik et al. 2008). Trypsin pretreatment of mitochondria, which removes TOM receptors, should abolish import, confirming receptor dependence (Kreimendahl et al. 2020).

Finally, functional specificity must be carefully validated. Hallmarks such as ΔΨm loss, cytochrome c release, or DRP1 activation are not unique to OMP-mediated damage and can arise from general stress. These readouts should therefore be paired with pathway-specific controls, including inactive mutants, blocking antibodies, or pharmacological rescue using caspase or DRP1 inhibitors. Phenotypes that depend on active OMPs and are reversible by such treatments provide the most convincing evidence of mechanism-specific mitochondrial subversion.

**Validation summary**. For each recombinant OMP or OMV experiment, best practice includes: (i) verification of lipopolysaccharide removal and specificity of effects; (ii) confirmation of proper folding and oligomerization by structural and biochemical assays; (iii) definition of a non-lytic concentration range using dose–response analysis; (iv) comprehensive OMV characterization and uptake mapping; (v) demonstration of mitochondrial fraction purity; (vi) proof of membrane integration via protease protection and alkaline extraction; and (vii) linkage of functional outcomes to active OMPs using mutant and rescue controls. Adhering to these criteria minimizes experimental artifacts and ensures robust, reproducible mechanistic conclusions on how bacterial OMPs and OMVs manipulate host mitochondria.

### Manipulation of mitochondrial function and apoptosis by β-barrel virulence factors

Pathogenic bacteria use diverse β-barrel OMPs to interact with host mitochondria and subvert their functions. These proteins can associate with or insert into mitochondrial membranes, disrupting homeostasis, modulating apoptotic signaling, and reshaping host metabolism to favor infection. The evolutionary conservation of β-barrel protein targeting is remarkable—fungal VDAC can assemble into the bacterial OM (Walther et al. 2010), and several bacterial β-barrel proteins, including PorB, PhoE, OmpA, Omp85, and OmpC, localize efficiently to the OMM in yeast and mammalian cells (Müller et al. 2002, Walther, Papic et al. 2009). Well-documented examples such as the OmpA/Omp38 protein of *A. baumannii*, OmpA of *Stenotrophomonas maltophilia*, and PorB of *N. gonorrhoeae* illustrate this cross-compartment compatibility and demonstrate how bacterial OMPs can exploit mitochondrial pathways to promote virulence, whereas similar effects for other Gram-negative OMPs remain speculative and require further experimental validation (Rumbo et al. 2014, Matsuo et al. 2017, You et al. 2020).

#### Acinet*obacter baumannii* OmpA

**Figure 2.**
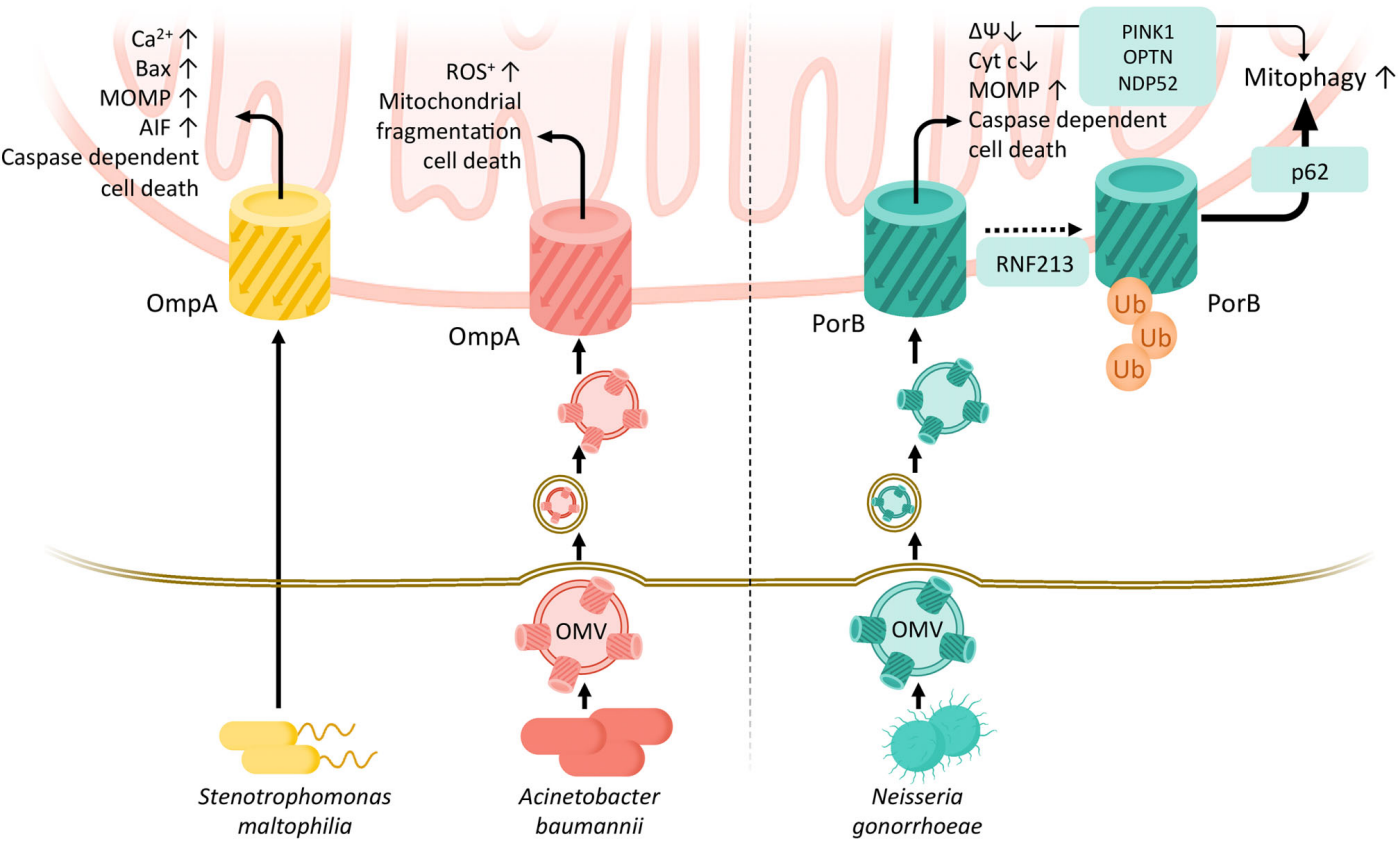
Manipulation of host mitochondrial functions by bacterial β-barrel virulence factors. OmpA from *S. maltophilia* or *A. baumannii* induces mitochondrial cell death when delivered to the host mitochondrial OM. PorB from *N. gonorrhoeae*, once inserted at mitochondrial membranes, can either induce apoptotic cell death or ΔΨm-mediated mitophagy via the PINK1 pathway or, after polyubiquitination through the RNF213 ubiquitin ligase, activate mitophagy via the p62 pathway. Endolysosomal uptake and escape within the host cell is depicted for the cases of known Omp delivery via OMVs. MOMP, mitochondrial outer membrane permeabilization; AiF, apoptosis-inducing factor; ROS, reactive oxygen species; Cyt *c*, cytochrome *c*; ΔΨm, mitochondrial membrane potential; OMV, outer membrane vesicle; Ub, ubiquitin.

*Acinetobacter baumannii* is an aerobic, pleomorphic Gram-negative bacterium recognized as a significant pathogen responsible for severe infections, including pneumonia and bloodstream infections, which often have fatal outcomes (Wong et al. 2017). Its high prevalence of multidrug-resistant phenotypes has led the World Health Organization to designate it as a “priority one pathogen”, emphasizing the urgent need for understanding its pathogenicity mechanisms. A key virulence factor of *A. baumannii* is outer membrane protein A (OmpA_Ab_​). Elevated expression of OmpA_Ab_ ​has been identified as a significant risk factor contributing to increased mortality in human infections caused by this pathogen (Sánchez–Encinales et al. 2017). OmpA_Ab_ shares homology with the monomeric OM protein A (OmpA) from *Enterobacteriaceae*, but lacks the trimeric structure typical of classical porins such as OmpC, OmpF, or LamB. (Gribun et al. 2003). Structurally, it features extracellular loops that extend outward and is non-covalently anchored to the peptidoglycan layer within the bacterial periplasm. Functionally, OmpA_Ab_ forms a non-selective channel in the bacterial OM, facilitating the translocation of ions and other solutes (Sugawara and Nikaido 2012). Beyond its role in permeability, OmpA_Ab_ plays multifaceted roles in *A. baumannii* pathogenesis, including promoting resistance to complement-mediated killing and supporting biofilm formation (Kim et al. 2009, Gaddy et al. 2009). Additionally, virulence factors such as OmpA_Ab_ and specific tissue-degrading enzymes are delivered to host cells via OMVs (Jin et al. 2011), further enhancing the pathogenic potential of this bacterium. Previous studies explored how apoptosis occurs in human laryngeal epithelial HEp-2 cells when infected with live *A. baumannii* or treated with purified OmpA_Ab_ protein. The findings revealed that OmpA_Ab_ targets mitochondria, prompting the release of cytochrome c and AIF into the cytosol, thereby triggering apoptosis through both caspase-dependent and AIF-dependent pathways (Choi et al. 2005) (Fig. 2). Recent work elucidates how *A. baumannii* employs OmpA_Ab_ to damage host cells during infection. Mechanistically, OmpA_Ab_ is delivered to host cells primarily through OMVs, which are internalized via endocytic uptake routes and cause mitochondrial fragmentation and cell toxicity (Tiku et al. 2021). Experimental evidence shows that even *E. coli* expressing OmpA_Ab_ triggers similar mitochondrial damage, confirming that OmpA_Ab_ alone is sufficient to induce this effect. Fluorescent detection of intact OmpA_Ab_-containing OMVs in infected cells confirmed evasion from lysosomal degradation and colocalization with mitochondrial structures (Tiku et al. 2021). It remains, however, to be determined whether and how OmpA_Ab_ is physically inserted into mitochondrial membranes to trigger mitochondrial toxicity. The mechanism involves OmpA_Ab_ activating the host GTPase protein DRP1, which associates with mitochondria and drives both mitochondrial fragmentation and cell toxicity. Disruption of DRP1 prevents these effects. While the majority of mechanistic data on OmpA_Ab_-induced apoptosis stems from immortalized epithelial models such as HEp-2 or HeLa cells, the relevance of these findings has now been confirmed *in vivo*. Infected mice display OmpA_Ab_-dependent mitochondrial fragmentation in alveolar macrophages, and OmpA_Ab_ delivery via OMVs was shown to enhance bacterial dissemination beyond the lung, emphasizing that this mechanism operates in primary immune cells under physiological conditions (Tiku et al. 2021). Taken together, the evidence supporting OmpA_Ab_ mitochondrial targeting and apoptotic activity is strong, as its delivery via OMVs has been demonstrated in epithelial cell cultures, with mitochondrial localization and cytotoxic effects further confirmed in murine infection models *in vivo* (Choi et al. 2005, Tiku et al. 2021). OMV-associated OmpA_Ab_ is emerging as a key contributor to *A. baumannii* pathogenicity and should be considered in the development of anti-infective therapies (Oh et al. 2025).

#### Stenotrophomonas *maltophilia* OmpA

*Stenotrophomonas maltophilia*, an obligate aerobic, non-fermenting Gram-negative bacterium, is widely found in nature and can reside in the human respiratory tract and feces. It is recognized as a common opportunistic pathogen, particularly in clinical settings, with its prevalence in intensive care units steadily increasing. Clinical isolates of *S. maltophilia* often exhibit high resistance to antimicrobial agents, such as aminoglycosides and β-lactams, posing significant therapeutic challenges. Previous studies investigated the role of *S. maltophilia* OmpA (OmpA_Sm_) in inducing apoptosis in HEp-2 epithelial cells using purified recombinant OmpA_Sm_ (Wang et al. 2020). Exposure to OmpA_Sm_ triggered apoptosis-related features such as nuclear rounding, chromatin condensation, and phosphatidylserine exposure. OmpA_Sm_ altered Bax and Bcl-xL protein levels, increasing mitochondrial permeability and releasing cytochrome c and apoptosis-inducing factors, which activated the caspase-9/caspase-3 pathway. Additionally, OmpA_Sm_ elevated ROS and intracellular Ca²⁺ levels. These findings indicate that OmpA_Sm_ induces apoptosis through mitochondrial pathways (Wang et al. 2020) (Fig. 2). To date, the apoptotic activity of OmpA_Sm_ has been characterized mainly in HEp-2 epithelial cell cultures, and further validation in primary or *in vivo* models is still lacking.

#### *Neisseria gonorrhoeae* PorB

*Neisseria gonorrhoeae* is an exclusively human pathogen that causes gonorrhea, a common sexually transmitted infection. To ensure its own persistence within the host, *N. gonorrhoeae* actively interferes with both the innate and adaptive arms of the immune system. Central to this process is PorB_Ng_, the most abundant OMP of *Neisseria*, which serves not only as an essential ion-conducting channel but also as a key immunomodulatory factor (Zeth et al. 2013). PorB_Ng_ undermines innate immunity by reducing the bactericidal activities of macrophages and neutrophils, and by attracting complement regulatory molecules to the bacterial surface, thereby diminishing complement-mediated killing. In terms of adaptive immunity, PorB_Ng_ impairs the ability of dendritic cells to drive T-cell proliferation, ultimately weakening the host capacity to mount an effective immune response (Jones, Jerse et al. 2024). Importantly, PorB_Ng_ has intricate modulatory effects on mitochondria-mediated apoptosis and mitophagy once internalized in human epithelial and immune cells. Exposure of epithelial or immune cells to purified PorB_Ng_ induces their apoptosis (Müller et al. 1999).

During infection, PorB_Ng_ migrates from the bacterial OM to host cell mitochondria, exhibiting nucleotide binding and voltage-dependent gating properties similar to human VDAC. This can be recapitulated with purified PorB_Ng_, which is efficiently and selectively imported into mitochondria of infected cells and blocking this translocation prevents apoptosis, indicating that PorB_Ng_ functions at the mitochondrial checkpoint of cell death (Müller et al. 2000). Initial import studies with endogenously expressed PorB_Ng_ in HeLa cells indicated a TOM dependent mitochondrial import and oligomeric insertion in the OMM (Müller et al. 2002). Both *in vitro* and *in vivo* exposure to PorB_Ng_ leads to cytochrome c depletion, mitochondrial permeability transitions, and morphological changes such as matrix condensation and cristae loss. Further biochemical import assays confirm true membrane integration: trypsin pretreatment of isolated mitochondria blocks PorB_Ng_ insertion, implicating TOM receptors, while alkaline carbonate extraction retains PorB_Ng_ in the protease-protected, membrane-embedded fraction. Yeast and mammalian models show that PorB_Ng_ import depends on TOM but not SAM, suggesting a distinct post-TOM insertion pathway to the inner mitochondrial membrane (Kozjak–Pavlovic et al. 2009). An ATP-binding–deficient PorB mutant cannot trigger ΔΨm loss or apoptosis, indicating that ΔΨm disruption is essential for PorB-induced cell death (Kozjak–Pavlovic et al. 2009).

More recent studies revealed that *N. gonorrhoeae* releases PorB_Ng_ through OMVs, which deliver the porin to host mitochondria. OMVs contain PorB_Ng_ in its native trimeric form and transfer it efficiently to macrophages and epithelial cells, as shown by immunofluorescence and immunoblotting (Deo et al. 2018). Following endocytic uptake and partial endolysosomal escape, PorB_Ng_-containing OMVs reach the cytosol and finally associate closely with the mitochondrial surface. PorB_Ng_ is then efficiently transferred to host mitochondrial membranes in its monomeric form as shown by cell fractionation experiments, while the mechanistic details of this transfer are still to be elucidated (Deo et al. 2018). Most mechanistic analyses of PorB_Ng_ mitochondrial targeting have been conducted in HeLa or macrophage-like cell lines, which are highly responsive to stress stimuli. However, PorB_Ng_ translocation and apoptotic effects have also been detected in primary human macrophages and epithelial explants, and OMV-mediated PorB_Ng_ delivery has been reproduced in murine infection models, lending physiological support to the mitochondrial manipulation mechanisms observed *in vitro* (Deo et al. 2018, Gao et al. 2024).

Although extensive studies have confirmed PorB_Ng_ targeting to host mitochondria, its precise intramitochondrial localization remains unresolved. While early biochemical and import analyses supported insertion into the IMM via the TOM complex, more recent data obtained from OMV delivery systems and electrophysiological studies point to a predominant association with the OMM. These differing results highlight that PorB_Ng_ mitochondrial topology remains a matter of active debate and may depend on the cellular context or mode of delivery (see Box 4 for a detailed discussion of the competing models and supporting evidence).

Box 4. Mitochondrial localization of *N. gonorrhoeae* PorB: still under debate

Although PorB_Ng_ is one of the best-characterized bacterial β-barrel proteins targeting mitochondria, its precise intramitochondrial localization remains controversial. Two principal models have emerged to explain its positioning and function within host cells. The first proposes that PorB is imported through the TOM complex and subsequently integrates into the IMM, where its channel activity directly collapses the membrane potential (ΔΨm) and initiates apoptosis. The second model argues that PorB_Ng_ associates with or partially inserts into the OMM, functioning analogously to the VDAC to mediate cytochrome c release and trigger cell death from the mitochondrial surface.

Early biochemical and import studies strongly supported the inner-membrane model. In these experiments, PorB_Ng_ remained associated with mitochondrial membranes after alkaline carbonate extraction, confirming genuine membrane integration, and was protected from protease digestion unless TOM receptors were blocked by trypsin pretreatment, indicating TOM-dependent import (Müller et al. 2000, 2002). Moreover, ATP-binding–deficient PorB_Ng_ mutants failed to induce ΔΨm loss, linking nucleotide binding and inner-membrane insertion to PorB_Ng_ apoptotic activity (Kozjak–Pavlovic et al. 2009). In contrast, structural and functional analyses using mitoplast electrophysiology and OMV delivery systems have provided evidence more consistent with outer-membrane localization. OMV-released PorB_Ng_ reaches mitochondria in its native trimeric form, associates with the organelle surface, and is subsequently detected in a monomeric state after transfer—findings that suggest partial insertion into the OMM rather than full translocation to the IMM (Deo et al. 2018).

These apparently conflicting results likely reflect the use of different experimental systems and import routes. It is conceivable that PorB_Ng_ initially associates with the OMM and, under certain conditions progresses through TOM to the IMM. For now, the dual capacity of PorB_Ng_ to interact with both mitochondrial membranes underscores its remarkable adaptability as a bacterial β-barrel effector that can exploit conserved host import machineries to subvert mitochondrial integrity.

Collectively, these findings demonstrate that PorB_Ng_ exploits conserved β-barrel import signals and the TOM complex to integrate into mitochondria, where its channel activity collapses ΔΨm, releases cytochrome c, activates caspases, and induces macrophage apoptosis—providing *Neisseria* with a means to subvert immune defenses (Deo et al. 2018) (Fig. 2).

Very recently, an intriguingly sophisticated mechanism was discovered, revealing how PorB_Ng_ manipulates additional mitochondrial functions, specifically mitophagy, in infected cells (Gao et al. 2024). Neisserial OMVs containing PorB_Ng_ prompt mitophagy in epithelial cells, thereby limiting mitochondrial ROS production and enhancing bacterial intracellular survival. Mechanistically, PorB-induced loss of mitochondrial membrane potential activates the PINK1–OPTN/NDP52 axis, initiating a classical mitophagy response. In parallel, PorB_Ng_ directly recruited the E3 ubiquitin ligase RNF213, which tagged a specific lysine residue (K171) on the porin with K63-linked polyubiquitin chains, initiating mitophagy in a p62-dependent manner (Fig. 2). Current evidence indicates that RNF213 acts independently of PINK1/Parkin, functioning as an alternative ubiquitin ligase rather than a cofactor, thus defining a non-canonical mitophagy pathway centered on PorB_Ng_-modified mitochondria. The findings highlight a mechanism by which the polyubiquitination of a bacterial β-barrel factor targeting mitochondria facilitates mitophagy, thereby mitigating ROS release and contributing to bacterial survival (Gao et al. 2024, Gao and van der Veen 2024). Taken together, PorB_Ng_ mitochondrial localization and pro-apoptotic effects have been demonstrated through OMV-mediated delivery, supported by extensive data from *in vitr o* and cell culture studies as well as validation in murine infection models, confirming its function under physiological conditions (Deo et al. 2018, Gao et al. 2024).

Table 1 provides an overview of the delivery routes and mitochondrial effects exerted by the OMP virulence factors discussed in this review.

**Table 1.** Delivery routes and mitochondrial manipulation mechanisms of OmpA and PorB.

Virulence factor	OmpA (*A. baumannii*)	OmpA (*S. maltophilia*)	PorB (*N. gonorrhoeae*)
Structure	8 stranded β-barrel with C-terminal peptidoglycan binding domain and variable extracellular loops	8 stranded β-barrel with C-terminal peptidoglycan binding domain and variable extracellular loops	16 stranded β-barrel with variable extracellular loops
Host cell delivery	OMVs → endocytic uptake	Not experimentally defined; recombinant protein exposure	OMVs → endocytic uptake
Mitochondrial entry pathway	OMV internalization → endocytic trafficking → partial endolysosomal escape → mitochondrial targeting mechanism not fully resolved	Likely direct mitochondrial association; import pathway unknown	– OMV internalization → endocytic trafficking → partial endolysosomal escape → translocation to mitochondria – TOM-dependent mitochondrial import (demonstrated with purified PorB); SAM-independent
Target compartment	Association with mitochondria; precise insertion into OMM vs. IMM unresolved.	Mitochondria (OM-associated dysfunction inferred)	OMM/IMM (context-dependent)
Cellular outcomes	– Mitochondrial fragmentation – Cyt c and AIF release – Caspase and AIF dependent apoptosis – DRP1-mediated fragmentation and cellular toxicity	– Cyt c and AIF release – Caspase-9/3 pathway activation – Apoptosis induction (nuclear rounding, chromatin condensation, phosphatidylserine exposure)	– ΔΨm collapse – Cyt c depletion – Caspase activation – mitophagy induction; macrophage apoptosis; ROS modulation
Experimental evidence	– HEp-2 epithelial cells infected with *A. baumannii* or treated with purified OmpA_Ab_ – Murine infection models	– HEp-2 epithelial cell cultures using recombinant OmpA_Sm_	– HeLa or macrophage-like cell lines – Primary human macrophages and epithelial explants – Murine infection models
Mechanistic motifs	– Host GTPase protein DRP1 activation – ROS increase	– Bax protein increase – MOMP increase – Elevated intracellular Ca²⁺ and ROS increase	– VDAC mimicry – PINK1/RNF213-mediated mitophagy – ROS modulation
Reference	(Choi et al. 2005, Jin et al. 2011, Tiku et al. 2021)	(Wang et al. 2020)	(Deo et al. 2018, Müller et al. 2000, 2002, Gao et al. 2024)

## OMP virulence factors as emerging antimicrobial targets

There are two primary approaches to developing new antimicrobial agents. The first involves blocking essential components or the production of substances critical for bacterial survival (Chen et al. 2019, Kawai et al. 2019), while the second targets virulence factors or antibiotic resistance genes to reduce pathogenicity or enhance sensitivity to existing antibiotics (Krueger and Brown 2019, Gadar and McCarthy 2023). However, targeting a single essential component often exerts significant evolutionary pressure on bacteria, accelerating the emergence of highly drug-resistant strains (Monserrat–Martinez et al. 2019). Consequently, innovative strategies that focus on non-essential processes have become crucial to overcoming bacterial resistance effectively (Mühlen and Dersch 2016, Gadar and McCarthy 2023).

Bacterial β-barrel OMPs are attractive intervention points because they are surface-exposed, central to virulence and mitochondrial subversion, and in several cases, immunogenic and sufficiently conserved to be targeted. Therapeutic strategies under exploration broadly fall into three classes: (i) vaccination against extracellular loops, (ii) direct inhibition of OMP function by peptides or small molecules, and (iii) anti-virulence approaches that interfere with OMV-mediated delivery of OMP cargo. Below we outline each strategy, with a focus on β-barrel OMPs implicated in mitochondrial manipulation.

### Vaccines directed against OMP surface loops

OmpA_Ab_ is one of the most intensively studied β-barrel OMPs as a vaccine antigen (Nie et al. 2020). OmpA_Ab_ is highly conserved across clinical isolates (>99% amino acid identity) and shows low similarity to human proteins (Luo et al. 2012), which reduces autoimmunity concerns. Immunization with recombinant OmpA_Ab_ or OmpA_Ab_-derived constructs elicits strong OmpA_Ab_-specific IgG responses, lowers bacterial burden in mouse infection models (up to 10-fold reductions in lung or bloodstream CFU), and improves survival following lethal challenge (Luo et al. 2012, Lin et al. 2013, Bolourchi et al. 2019). Similar work has explored PorB_Ng_ as a vaccine component or DNA vaccine antigen (Zhu et al. 2004, Maurakis and Cornelissen 2022, Viviani et al. 2023).

Two caveats are emerging. First, extracellular loops of OMPs can be variable, especially in *Neisseria* (Derrick et al. 1999, Bennett et al. 2008), which may limit cross-strain protection and drive immune escape. Second, PorB_Ng_ can itself modulate host immunity in immunosuppressive ways, which complicates its straightforward use as a vaccine antigen (Jones, Jerse et al. 2024, Jones, Ramirez–Bencomo et al. 2024). Newer approaches—including multiepitope or mRNA-based OmpA_Ab_ vaccines (Negahdari et al. 2024, Ma et al. 2025)—aim to present conserved loop epitopes while minimizing unwanted immunomodulation.

### Peptide and small-molecule inhibitors of OMP function

A complementary strategy is to block OMP activity directly, rather than eliciting immunity. For *A. baumannii*, therapeutic peptides that bind OmpA_Ab_ and interfere with its function have shown efficacy *in vitro* and in animal infection models (Vila–Farrés et al. 2017, Parra-Millán et al. 2018, Zhao et al. 2024). These inhibitors can sensitize multidrug-resistant strains, reduce serum resistance, and synergize with last-line antibiotics such as colistin, effectively restoring killing even in colistin-resistant isolates (Parra–Millán et al. 2018). In addition, small-molecule or antisense/translation-blocking strategies that down-regulate OmpA_Ab_ expression have been reported to reduce *A. baumannii* virulence *in vivo* (Chen et al. 2019, Seok–Hyeon Na et al. 2021, Seok Hyeon Na et al. 2021).

Conceptually, PorB_Ng_ represents a similar target class: because PorB_Ng_ can integrate into host mitochondria and collapse ΔΨm, direct PorB_Ng_ blockers (e.g. loop-binding peptides or channel blockers) could, in principle, prevent mitochondrial injury during infection. However, this strategy carries a specific safety concern: PorB_Ng_ forms a voltage-dependent β-barrel with properties reminiscent of the host VDAC channel in the OMM. Any small molecule designed to inhibit PorB_Ng_ conductance must be evaluated for off-target interference with VDAC, because broad VDAC blockade could dysregulate ion flux, metabolism, and apoptosis in host tissues (Shoshan–Barmatz et al. 2020). For that reason, PorB_Ng_-directed channel inhibitors are mechanistically attractive but will likely face a higher translational barrier than OmpA_Ab_-directed inhibitors, where the main risk is resistance, not host-channel cross-reactivity.

### Anti-virulence strategies targeting OMV delivery

The β-barrel OMPs discussed in this review—including OmpA_Ab_ and PorB_Ng_—are not only surface proteins but are also packaged into OMVs, which act as delivery vehicles to host cells and, in some cases, to host mitochondria (Jin et al. 2011, Deo et al. 2018, Tiku et al. 2021, Gao et al. 2024). Because OMVs concentrate OMPs in a native lipid environment, protect them from degradation, and traffic them into host cells via endocytic uptake, OMV production and uptake are attractive anti-virulence targets.

Three intervention concepts can be explored in the field:

(i) Inhibiting OMV biogenesis or release, to reduce delivery of toxic OMP cargo. Genetic or chemical disruption of OMV formation can attenuate virulence phenotypes in several Gram-negative pathogens *in vitro*, though broad-spectrum OMV inhibitors suitable for therapeutic use in humans are not yet established. (ii) Neutralizing OMVs extracellularly, e.g. using antibodies that bind abundant OMP epitopes exposed on OMVs, thereby blocking uptake into host cells. In principle, OmpA_Ab_ or PorB_Ng_ loop-directed antibodies could function this way, acting more like toxin-neutralizing antibodies than classical opsonizing antibodies (Lei et al. 2024, Oh et al. 2025). (iii) Decoy strategies, in which synthetic vesicles or liposomes are engineered to absorb OMVs or sequester their OMP cargo before it reaches target host cells. This approach aims to blunt mitochondrial damage and inflammatory signaling without directly killing the bacterium, thereby applying less evolutionary pressure for antibiotic resistance (Mühlen and Dersch 2016, Krueger and Brown 2019).

### Translational outlook: opportunities and hurdles

From a product-development perspective, these three strategies occupy different risk zones. Vaccines targeting conserved OMP loops (e.g. OmpA_Ab_) have already shown protection in animal models, are compatible with mRNA or multiepitope platforms, and could in principle be advanced toward prophylaxis in high-risk hospital settings. In contrast, direct PorB_Ng_ or OmpA_Ab_ functional inhibitors resemble anti-toxin biologics but must balance potency with selectivity, especially in the case of PorB_Ng_, to avoid interfering with host mitochondrial channels such as VDAC. OMV-directed anti-virulence interventions are conceptually attractive—they aim to disarm rather than kill—but will require demonstration that reducing OMP-loaded OMV delivery meaningfully alters disease course *in vivo* and does not simply shift the bacterium toward alternative secretion strategies.

Overall, β-barrel OMPs represent a compelling new class of intervention targets at the host–pathogen interface. However, successful clinical translation will require (i) antigen designs that accommodate surface loop variability and immune evasion, (ii) inhibitors that avoid mitochondrial off-target liabilities, and (iii) anti-virulence approaches that achieve meaningful benefit without driving rapid compensatory resistance. These considerations define the realistic boundaries for future therapeutic exploration, complementing the mechanistic insights summarized throughout this review.

## Concluding remarks

The biological functions of bacterial OMP virulence factors extend far beyond structural roles, revealing an unexpectedly sophisticated ability to manipulate host cell processes. Among the most striking aspects of OMP-mediated pathogenesis is their capacity to hijack host cell metabolism and, in particular, mitochondrial function. By exploiting the evolutionary link between mitochondria and their bacterial ancestors, OMPs subvert essential cellular mechanisms, triggering apoptosis, altering immune responses, and even modulating mitophagy to enhance bacterial survival. As research continues to uncover the molecular mechanisms underlying OMP-mediated virulence, it is becoming increasingly evident that these proteins play highly specialized roles in host–pathogen interactions. Future studies will likely reveal additional pathways through which OMPs interfere with host cell homeostasis, shedding light on previously unrecognized bacterial survival strategies. A deeper understanding of these mechanisms is essential for developing new therapeutic strategies. Targeting OMPs, whether through vaccines, inhibitors, or other antimicrobial approaches, holds great potential in combating bacterial infections, particularly those caused by multidrug-resistant Gram-negative pathogens. By integrating molecular insights with innovative treatment approaches, we can move closer to effective interventions that disrupt bacterial virulence without exacerbating antibiotic resistance.
